# Estimated effects of the implementation of the Mexican warning labels regulation on the use of health and nutrition claims on packaged foods

**DOI:** 10.1186/s12966-021-01148-1

**Published:** 2021-06-10

**Authors:** Carlos Cruz-Casarrubias, Lizbeth Tolentino-Mayo, Stefanie Vandevijvere, Simón Barquera

**Affiliations:** 1 Centro de Investigación en Nutrición y Salud, Instituto Nacional de Salud Pública, Universidad No. 655, Colonia Santa María Ahuacatitlán, Cerrada Los Pinos y Caminera. C.P, Cuernavaca, Morelos 62100 Mexico; 2 Department of Epidemiology and Statistics, School of Population health, The University of Auckland, Auckland, New Zealand

**Keywords:** Ultra-processed foods, Nutrient profile, Health claims, Nutrition claims

## Abstract

**Background:**

The use of health and nutrition claims on front-of-pack labels may impact consumers’ food choices; therefore, many countries have established regulations to avoid misinformation. This study describes the prevalence of health and nutrition claims on the front-of-pack of food products in retail stores in Mexico and estimate the potential effects of the Official Mexican Standards 051 (new regulation that includes specifications for implementing warning labels and other packaging elements such as health and nutrition claims on less healthy foods) on the prevalence of these claims.

**Methods:**

This is a cross-sectional study in which health and nutrition claims, nutrition information panels, and the list of ingredients of all foods and beverages available in the main retail stores in Mexico City were collected. The products were grouped by level of processing according to the NOVA food system classification. Claims were classified using the internationally harmonized INFORMAS taxonomy. According to the criteria of the new Mexican front-of-pack labelling regulation, the effect on the reduction on the prevalence of health and nutrition claims was estimated by type of food and by energy and nutrients of concern thresholds.

**Results:**

Of 17,264 products, 33.8% displayed nutrition claims and 3.4% health claims. In total, 80.8% of all products in the Mexican market were classified as “less healthy”; 48.2% of products had excess calories, 44.6% had excess sodium, and 40.7% excess free sugars. The new regulation would prevent 39.4% of products with claims from displaying health and nutrition claims (*P* < 0.001); the largest reduction is observed for ultra-processed foods (51.1%, *P* < 0.001). The regulation thresholds that resulted in the largest reduction of claims were calories (OR 0.62, *P* < 0.001) and non-sugar sweeteners (OR 0.54, *P* < 0.001).

**Conclusions:**

The new Mexican front-of-pack labelling regulation will prevent most processed and ultra-processed foods from displaying health and nutrition claims and will potentially improve information on packaging for consumers.

**Supplementary Information:**

The online version contains supplementary material available at 10.1186/s12966-021-01148-1.

## Background

Non-communicable diseases (NCDs) contribute to more than half of the global burden of disease and unhealthy diets are one of the main risk factors for ill health [1]. In 2017, it was estimated that 11 million deaths worldwide (22% of all deaths among adults), mainly those caused by NCDs, such as cardiovascular disease, cancers and type 2 diabetes, were attributable to unhealthy diets [2]. According to the last Mexican National Health and Nutrition Survey (ENSANUT by its acronym in Spanish), between 2012 and 2018 there was an increase in the prevalence of overweight and obesity in children (34.8 to 35.6%), adolescents (34.9 to 38.4%), and adults (71.3 to 75.2%) [3]. The proportion of adults with type 2 diabetes also increased from 6.4 million (9.2%) in 2012 to 8.6 million (10.3%) in 2018 [3]. Diabetes is the main cause of disability and the third cause of mortality in Mexico [4].

The main dietary components associated with increased risk for NCDs are a high intake of sodium, added sugars, and saturated fats [5]; high levels of these nutrients are commonly found in ultra-processed foods and beverages [6, 7]. According to the NOVA food classification (a system that categorizes foods according to the nature, extent and purpose of food processing, rather than by nutrients [8]), ultra-processed foods are ingredient formulations that result from a series of industrial processes. They generally include the addition of sugars, fats, sodium, and additives such as colorants, flavors, texturizers, humectants, and others to make them hyperpalatable; some examples are carbonated drinks, breakfast cereals, and instant soups [9]. In the Mexican population, added sugars and saturated fats contribute 12.5 and 11.2% (respectively) to total energy intake [10]. At the same time, the intake of these nutrients of concern rises with increased consumption of ultra-processed foods and beverages [10]. The World Health Organization (WHO) recommends that free sugars should contribute less than 10% of total energy intake to prevent NCDs and ideally less than 5% for additional health benefits [11].

Improved nutrition labelling (a description intended to inform the consumer about a food’s nutritional properties) is a strategy recommended by WHO and the Pan American Health Organization (PAHO) to prevent NCDs, with the primary purpose of helping consumers make healthier food choices [12]. Nutrition labelling on the back-of-pack is often ignored by consumers or can be confusing as they generally prefer shorter and simpler messages on the front-of-pack [13, 14]. Front-of-pack labelling (FOPL) has been shown to help consumers make healthier food choices [15, 16]. The most commonly used schemes to date include warning labels, traffic lights, the Nutri-score, and the Health Star Ratings.

Health claims (“any presentation that states, suggests or implies a relationship between a food or a constituent of that food and health”) and nutrition claims (“any presentation that states, suggests or implies that a food has particular nutritional properties, including but not limited to the energy value and to the content of protein, fat and carbohydrates, as well the content of vitamins and minerals”) are also ways of presenting health-related product information to consumers [17]. However, such claims constitute a form of advertising on packaging that can influence consumers’ purchases (e.g. claims and product information can motivate consumers’ purchasing decisions [18]), preferences (e.g. consumers interested in their health prefer products displaying health and nutrition claims (HNC) [19]), and/or consumption (e.g. nutrition claims can lead consumers to a higher energy intake because they perceive them as low calorie products [20]). Packaging displaying HNC can generate “health halos” making products look healthier regardless of their nutritional quality [21]. This can mislead consumers, who may misinterpret the nutritional quality of products with HNC [13, 22].

Research in various countries (e.g. New Zealand [23], United Kingdom [24], Ireland [25], and Brazil [26]) shows that for some food categories (e.g. cereals, beverages and dairy products), more than half of the products display HNC on packaging. These studies have shown that products displaying HNC tend to have a more favorable nutritional profile compared to those without HNC, although these differences were not always statistically significant. Moreover, it has been observed that in some food categories, less-healthy food products carry HNC more frequently than healthier foods [23]. Limiting the use of HNC on less-healthy foods is part of FOPL public policies. For this reason, it is important to monitor health-related labelling on food products to evaluate compliance with these policies [27]. Therefore, the International Network of Food and Obesity, NCDs Research, Monitoring and Action Support (INFORMAS) developed specific protocols for monitoring different types of HNC on food products, using a common taxonomy and harmonized methodology across countries [27, 28].

Some regions and countries in the world such as Australia and New Zealand have adopted specific regulations for the use of HNC on food products, including a series of nutritional criteria/thresholds that foods must pass in order to carry HNC [29]. However, nutrient profile models (algorithms for classifying foods and beverages according to their nutritional composition to promote public health dietary goals [30]) that are designed to prevent less-healthy food products from carrying claims only apply to health claims, not to nutrition claims, which are generally more common [29]. In March 2020, the Mexican FOPL system “Guideline Daily Amounts (GDA)”, which has been shown to be very confusing for consumers [31], was replaced by a warning label system. This label system is mandatory and includes warnings for calories, added sugar, saturated fat, trans fat and sodium [32]. This regulation went into effect in October 2020, although the thresholds for energy and nutrients of concern will become progressively stricter over 5 years. The specifications of the new regulation are found in the Official Mexican Standards 051 (NOM-051 for its acronym in Spanish), and aims to improve the information located on packaging for consumers; in addition, other elements such as advertising directed at children and the use of HNC on less-healthy foods and beverages will be regulated beginning in July 2021.

Unlike other countries that have implemented warning labels to date, such as Chile, Peru and Uruguay, the Mexican warning label system includes statements about certain additives that are not recommended for children, such as non-sugar sweeteners (artificial or natural non-caloric sweeteners or caloric sweeteners like polyols) and caffeine. In addition, the nutrient profile used by the Mexican regulation is based on the PAHO Nutrient Profile Model, which is more restrictive than the nutrient profile model used by Chile, Peru and Uruguay as it classifies all ultra-processed food products as less-healthy [33]. Food products with at least one warning label or warning legend must not display HNC on the front-of-pack.

This new public policy could prevent most of less-healthy packaged foods and beverages (specially ultra-processed foods) from including misleading information for consumers. However, it is unclear what impact the new Mexican FOPL regulation would have on the prevalence of HNC on less-healthy products.

The objectives of this study were to comprehensively measure the prevalence of HNC on the front-of-pack of foods and beverage products in the Mexican market and to estimate the potential effects of the new regulation on the prevalence of HNC on processed and ultra-processed food products.

## Methods

This is a cross-sectional study that analyzed the prevalence of HNC on the front-of-pack of foods and beverages available in retail stores in Mexico City during the period of January to March 2017. The retail stores were selected randomly and included the biggest supermarket chains in Mexico (such as the Walmart group, La Comer, Soriana, and Chedraui) and other types of retail stores. The selection of retail stores was made according to the urban Basic Geostatistics Areas (AGEB by its acronym in Spanish; it is a geographical area delimited by streets, avenues, walkways, or any other feature whose land use is dedicated to living, industrial, or commercial usage and its population is greater than 2500 inhabitants). The selection of AGEBs was determined according to the level of marginalization defined by the National Institute of Statistics and Geography (a Mexican sociodemographic indicator that considers the infrastructure for access to basic household services, health services, and education to measure the social disadvantages of AGEBs in three levels: low, middle, and high [34]) and population density (> 20,000 inhabitants). The selection of retail stores in each AGEB were selected randomly and proportionate to size.

Data were collected from 136 retail stores of different types: supermarkets (*n* = 52), price club (*n* = 8, a type of supermarket where membership is required, and which generally offers products contained in multi-packages for consumers and other establishments), wineries (*n* = 32), convenience stores (*n* = 20), mini supermarkets (*n* = 17), and other types of retail stores (*n* = 7). The selected retail stores included supermarket chains with more than 70% of the market share in Mexico [35].

To collect data, nutrition undergraduate students were trained by researchers from the National Institute of Public Health (INSP). Photographs were taken of each side of the package with a smartphone. When a product had a singular shape (cylinder, sphere or bag), the fieldworkers captured all relevant information. The following information was captured: Name of the product, the front of the package (including HNC), the type of package, GDA labelling, bar code, list of ingredients, nutrient facts table, and price. The fieldworkers walked through all the aisles of the retail stores to capture all products available (except products that were repeated/duplicated in different stores). Bar codes were used to identify duplicate products. Each field worker collected information on the same category of products from all included retail stores. Before taking photographs, we consulted the legal representatives of each store and/or the manager in charge for authorization.

Relevant information on food and beverage packages (as detailed below) was captured in REDCaP (Research Electronic Data Capture, an application for management of electronic data) by eight previously trained research assistants and exported in electronic spreadsheets.

### Health and nutrition claims

All claims that appeared on FOPL were registered and classified according to the INFORMAS protocols and taxonomy (Table 1) [27, 28]. The classification included three categories and their subcategories (see Additional file 1): 1) Nutrition claims (health-related ingredients claim, nutrient content claim, and nutrient comparative), 2) Health claims (general health claim, nutrient and other function claim, and reduction of disease risk claim), and 3) Other claims (i.e. organic, gluten free). The format of each claim was also recorded as verbal, numerical or symbolic. Products containing combinations of numerical and verbal formats were registered as numerical.

**Table 1:** Examples of different types of health and nutrition claims according to the INFORMAS taxonomy [27, 28]

Claim	Types of claims	Examples
Nutrition claim	Health-related ingredient claim	“Contains 25% orange juice” “Contains whole grains”
	Nutrient content claim	“Low calories” “Contains calcium”
	Nutrient comparative claim	“Light” “Sweetened with stevia”
Health claim	General health claim	“Low glycemic index” “Healthy”
	Nutrient and other function claim	“Calcium for strong bones” “Magnesium for growth”
	Reduction of disease risk claim	“Pediatric heart association” “Prevents cavities”
Other claim	Other claim	“Organic” “Non-GMO”

*INFORMAS* International Network for Food and Obesity/non-communicable Diseases Research, Monitoring and Action Support. *GMO* Genetically Modified Organisms

### Food groups

Food and beverages were grouped according to their degree of processing according to the NOVA classification system (a system to classify food and beverages according to the extent and purpose of food processing); this allows us to identify ultra-processed foods, which are commonly targeted in regulations related to FOPL, taxes and advertising directed to children. Food and beverages were classified as unprocessed or minimally processed foods (such as fresh fruits or vegetables, whole grain cereals, plain milk, and seeds with no added ingredients), processed culinary ingredients (such as salt, sugar or oil), processed foods (such as canned fruits and vegetables, salted seeds or meat with salt for preservation), and ultra-processed foods (such as carbonated beverages, ready to eat foods like pizza or hamburgers, pastries and breakfast cereals) [8]. The classification was made using the information available in the nutrition information panel and list of ingredients such as sugars, sodium, fat and added sweeteners, and others like emulsifiers, preservatives, binders, humectants, stabilizers, brighteners, colorants, and flavorings.

### Nutritional quality

Nutritional quality of the food products was calculated using the nutrient profile criteria of the Mexican FOPL regulation. Energy information was reported in calories, saturated fats, trans fats, and free sugars (such as sugar, sucrose, fructose, corn syrup, honey, and fruit juice) in grams per 100 g/mL, sodium in milligrams per 100 g/mL, and use of non-sugar sweeteners reported in the list of ingredients. For products that require preparation prior to consumption, the reconstituted content was considered. This nutrient profile is applicable to products containing added sugar, sodium or fat (*n* = 14,191), so unprocessed or minimally processed foods and processed culinary ingredients were automatically classified without excess nutrients of concern (healthier). To establish the cut-off points, the calories per gram were calculated for free sugars (4 kcal), saturated fats (9 kcal), and trans fats (9 kcal). The following criteria of the Mexican FOPL regulation were applied [32]: Excess of: a) calories: ≥ 275 kcal per 100 g for foods, ≥ 70 kcal per 100 mL for beverages or ≥ 8 kcal per 100 mL from free sugars for beverages; b) free sugars: ≥ 10% of total energy from free sugars; c) saturated fat: ≥10% of total energy from saturated fat; d) trans fat: ≥ 1% of total energy from trans fat; e) sodium: ≥1 mg of sodium per 1 kcal or ≥ 300 mg per 100 g, ≥ 45 mg per 100 mL for non-caloric beverages; and use of non-sugar sweeteners: reported in list of ingredients. For products without disaggregated content of free sugars on the package, we calculated free sugars according to the algorithms proposed by the PAHO nutrient profile model (see Additional file 2) [5].

### Scenarios for the use of health and nutrition claims

Two scenarios were applied to show the differences in the use of HNC before and after the implementation of the regulation.

The first scenario (current scenario) analyzes the prevalence of HNC on packaged food products in Mexico in 2017. The second scenario (regulatory scenario) was established following the specifications of the third and final stage of the new FOPL regulation in Mexico. The regulation requires that products with warning labels (excessive in calories, free sugars, saturated fats, trans fats and sodium) or warning legends (contains non-sugar sweeteners or added caffeine) must not [32]: a) use health claims, b) use nutrition claims, or c) display nutrition claims on the FOPL.

The following types of claims are considered in the regulation: Nutrient content claim, nutrient comparative claim, nutrient and other function claim, and reduction of disease risk claim. Consequently, these types of claims were covered in the analyses for the second scenario (Table 5 and Table 6). For health-related ingredient claims and general health claims (as per the INFORMAS taxonomy), the Mexican regulations do not apply.

We used the nutrient profile criteria of the Mexican FOPL regulation to determine which products are still allowed to display HNC.

### Statistical analysis

The analyses were performed using the statistical package STATA version 14. To verify the consistency in the classification of claims, we performed a reliability test between two raters (Table 2). A random sample of products that contained claims (*n* = 436) was taken and claims were classified according to the content and format by the two raters. The proportions of claims classified by category and subcategories were compared. The consistency in the reliability tests was determined using the Kappa Coefficient; values above 0.8 indicate very good consistency. The variables included in the analysis correspond to the categorical type, so they were presented as frequencies and percentages. The use of HNC was presented by food group, claim type (Table 4), and nutrient profile (Table 6). Chi-square tests were used to determine differences in the proportion of products with HNC between the current scenario and the regulatory scenario (Table 5 and Table 6). Two logistic regression models were fitted to determine the Odds Ratio (OR) of HNC prevalence in the current scenario and the regulatory scenario. Both models were adjusted for the components of the Mexican FOPL regulation and we report the results for each threshold of the nutrient profile separately. The analysis that included the regulatory scenario only considered products with claims (*n* = 8746). For all tests, the value *P* < 0.05 was considered as statistically significant.

**Table 2:** Inter-rater reliability for the classification of claims according to the INFORMAS taxonomy (*n* = 436)

	Rater 1		Rater 2		*P*-Value	K
	n	%	n	%		
Health-related ingredient claim	60	13.8	61	14.0	0.992	0.8744
Nutrient content claim	158	36.2	159	36.5	0.955	0.9314
Nutrient comparative claim	40	9.2	44	10.1	0.889	0.9039
General health claim	21	4.8	20	4.6	0.975	0.8775
Nutrient and other function claim	4	0.9	3	0.7	–	–
Reduction of disease risk claim	11	2.5	12	2.8	–	–
Environmental	245	56.2	247	56.7	0.911	0.9656
Other	163	37.4	165	37.8	0.940	0.9494
Numerical	78	17.9	99	22.7	0.433	0.8163
Verbal	211	48.4	216	49.5	0.820	0.8258
Symbolic	335	76.8	329	75.5	0.694	0.8743

*P*-value for difference between two proportions. K Cohen’s Kappa Coefficient

## Results

In general, there were no statistically significant differences between both raters in the proportion of each type of claim classified by food category and subcategory (*P* > 0.05). There was good agreement between raters for classification by categories and subcategories of claims (K > 0.8) (Table 2).

Photographs of 18,558 unique products were collected. Products contained in multi-packages (*n* = 533) and those with inconsistencies in nutritional information (*n* = 761) were excluded (for example, differences of more than 15% between reported and calculated calorie content, portion size, and sum of nutrients and units of nutrients). In total, 17,264 food and beverage packages available in retail stores in Mexico were included, of which, 72% were classified as ultra-processed foods, 10.4% as unprocessed or minimally processed foods, 9.9% as processed foods, and 7.4% as processed culinary ingredients. When evaluating the nutritional quality of all included products, 48.2% of food products were excessive in calories, 44.6% were excessive in sodium, and 40.7% were excessive in free sugars according to the thresholds of the Mexican FOPL regulation. For processed foods, 69.2% were excessive in sodium and 41.4% were excessive in calories; most of the ultra-processed foods were excessive in calories (61.0%) and free sugars (53.4%) (Table 3).

**Table 3:** Proportion of label components, claims and nutrient profile (*n* = 17,264), the Mexican food supply, 2017

	Total		Unprocessed or minimally processed foods (*n* = 1794)		Processed culinary ingredients (*n* = 1279)		Processed foods (*n* = 1705)		Ultra-processed foods (*n* = 12,486)	
	n	%	n	%	n	%	n	%	n	%
**Mexican FOPL nutrient profile**										
Passes nutrient profile	3403	19.7	1794	100	1279	100	88	5.2	242	1.9
Excessive in one or more nutrients of concern or energy	13,861	80.3	0	0	0	0	1617	94.8	12,244	98.1
Excessive in calories	8327	48.2	0	0	0	0	706	41.4	7621	61.0
Excessive in free sugars	7020	40.7	0	0	0	0	351	20.6	6669	53.4
Excessive in sodium	7699	44.6	0	0	0	0	118	69.2	6519	52.2
Excessive in saturated fats	6054	36.1	0	0	0	0	518	32.4	5536	45.8
Excessive in trans fats	156	2.1	0	0	0	0	17	4.1	139	3.7
Containing non-sugar sweeteners	2176	12.6	0	0	0	0	0	0	2176	17.4
**Label Components**										
List of ingredients	16,745	97.0	1472	82.1	1181	92.3	1659	97.4	12,433	99.6
Nutrient declarations	17,242	99.8	1793	99.9	1279	100	1705	100	12,465	99.8
**Supplementary nutrition information**										
GDA	13,598	78.8	1215	67.7	882	69	1359	79.7	10,142	81.2
*Sello Nutrimental*	18	0.1	3	0.2	6	0.5	2	0.1	7	0.1
**Use of claims**										
Yes	8746	50.7	1036	57.8	598	46.8	673	39.5	6439	51.6
No	8518	49.3	758	42.2	681	53.2	1032	60.5	6047	48.4
**Nutrition claim**	5839	33.8	746	41.6	296	23.1	299	17.5	4498	36.0
Health-related ingredient claim	1614	9.4	209	11.7	124	9.7	59	3.5	1222	9.8
Nutrient content claim	4667	27.0	660	36.8	184	14.4	247	14.5	3576	28.6
Nutrient comparative claim	998	5.8	45	2.5	61	4.8	27	1.6	865	6.9
**Health claims**	617	3.4	74	4.1	80	6.3	22	1.3	441	3.5
General health claim	306	1.8	60	3.3	68	5.3	12	0.7	166	1.3
Nutrient and other function claim	145	0.8	4	0.2	7	0.6	6	0.4	128	1.0
Reduction of disease risk claim	214	1.2	12	0.7	14	1.1	4	0.2	184	1.5
**Other claim**	5190	30.1	616	34.3	476	37.2	553	32.4	3545	28.4

*GDA* Guide Daily Amount. *Sello Nutrimental* is a type of voluntary labelling to indicate that food and beverages are healthy

More than half of all products (50.7%) displayed claims on the FOPL. Nutrition claims were the most frequent type of claims (33.8%), mainly nutrient content claims (27%). In terms of claim format, symbolic claims were the most frequently used (33.9%). Table 4 shows the proportion of products with claims by food group. The category of unprocessed or minimally processed foods had the highest proportion of products with claims, mainly plain milk (97.4%), juices and fruit drinks (77.1%), and purees of fruits, vegetables and cereals (76.4%). More than half of the ultra-processed food products displayed at least one claim (51.6%); claims were found most frequently for baby food (92.2%), breakfast cereals (82.2%), and yogurt and milk-based beverages (67.5%). Among products with claims, nutrition claims were common for unprocessed or minimally processed (72%) and ultra-processed foods (69.9%).

**Table 4:** Proportion of packaged food products with claims according to the NOVA food groups

	n	Use of Claims (%)	Nutrition claims (%)	Health Claims (%)	Other claims (%)
**Total**	17,264	50.7	66.8	7.1	59.3
**Unprocessed or minimally processed foods**	1794	57.8	72.0	7.1	59.5
Milk	113	97.4	100	10.9	36.4
Cereals	218	46.8	66.7	8.8	75.5
Fruits and vegetables	277	54.5	37.1	17.2	76.8
Legumes	87	9.2	62.5	0	37.5
Coffee and tea	135	62.2	57.1	7.1	71.4
Eggs, read meat and seafood	90	50.0	40.0	4.4	91.1
Water	109	51.4	85.7	14.3	30.4
Juices and fruit drinks	166	77.1	85.9	0.8	71.9
Nuts and seeds	126	61.9	74.4	3.9	73.1
Pastas	401	54.6	81.3	3.2	34.3
Puree of fruits, vegetables and cereals	72	76.4	85.5	0	69.1
**Processed culinary ingredients**	1279	46.8	49.5	13.4	79.6
Oils and fats	336	45.5	62.8	17.7	62.8
Dressings	315	51.8	34.4	13.5	92.0
Condiments	183	27.9	52.9	5.9	76.5
Sweeteners	445	51.9	50.7	12.1	82.7
**Processed foods**	1705	39.5	44.4	3.3	82.2
Fruits and vegetables (canned)	526	39.5	13.5	2.9	94.7
Meat and seafood	298	41.3	69.9	3.3	75.6
Sweet snacks	152	28.3	46.5	9.3	69.8
Salty snacks	348	35.6	65.3	1.6	71.0
Bread and other cereals	152	56.6	66.3	7.0	76.7
Cheeses	128	29.7	47.4	0	76.3
Other^a^	101	50.5	17.7	0	98.0
**Ultra-processed foods**	12,486	51.6	69.9	6.9	55.1
Yogurt and milk-based beverages	935	67.5	91.6	10.9	34.4
Ultra-processed meat	649	67.3	29.5	3.9	98.4
Breakfast cereals	555	82.2	83.8	11.0	48.7
Seafood	208	36.1	81.3	9.3	50.7
Beverages with non-sugar sweeteners^b^	948	67.5	96.6	8.8	19.2
Sugar-Sweetened beverages^c^	775	54.1	74.0	10.0	49.2
Sweet snacks^d^	3956	44.2	67.7	3.0	57.7
Salty snacks	912	35.8	58.6	2.8	70.6
Packaged bread and tortilla	215	65.6	75.2	8.5	46.1
Cheeses	360	31.9	64.4	7.0	52.2
Ready to eat	409	40.3	37.0	3.0	87.9
Baby food	218	92.2	88.1	13.9	39.8
Soups pastas and creams	326	60.4	50.8	1.0	71.6
Other^e^	2020	43.9	59.4	9.4	65.2

The percentages for nutrition claims, health claims and other claims represent a proportion from products that use claims. ^a^ Includes canned beans and prepared salads, ^b^ includes beverages sweetened with artificial or natural non-caloric sweeteners or polyols, ^c^ Includes nectars, fruit drinks with added sugar, energy drinks, sport drinks and powder to prepare beverages, ^d^ Includes candies, sweets, desserts and bakery, ^e^ Includes prepared flour bakery, prepared cereals, non-sugar sweeteners and ultra-processed culinary ingredients (margarine, seasonings for meat)

In the current scenario, 68.4% (95% CI 67.4–69.3) of all products and 71.0% (95% CI 69.8–72.1) of ultra-processed foods displayed HNC on the front-of-package (Table 5). This proportion is highest for beverages with non-sugar sweeteners (96.7, 95% CI 95.0–98.0) and baby food (93.0, 95% CI 88.6–96.1). In the regulatory scenario, the proportion of all products that would display HNC is significantly lower (39.4%, *P* < 0.001, 95% CI 38.1–40.8) compared to the current scenario. The differences were statistically significant for all food and beverage categories. The largest reduction in the prevalence of HNC was observed for ultra-processed foods, where the regulatory scenario would prevent 51.1% (*P* < 0.001, 95% CI 49.5–52.5) of these products from displaying HNC. Within the category of ultra-processed products, the highest reduction in the prevalence of HNC was observed in beverages with non-sugar sweeteners (85.9%, *P* < 0.001, 95% CI 82.3–88.7).

**Table 5:** Difference between current scenario and regulatory scenario for the prevalence of health and nutrition claims (*n* = 8746)

		Proportion of products with health and nutrition claims				Difference	
		Current scenario		Regulatory scenario			
	n	%	95% CI	%	95% CI	%	95% CI
**All products with claims**	8746	68.4	[67.4, 69.3]	28	[27.1, 29.0]	39.4***	[38.1, 40.8]
**Processed and ultra-processed foods**	7112	68.6	[67.3, 69.9]	19.1	[17.0, 21.2]	49.5***	[47.1, 51.2]
**Processed foods**	673	46.2	[42.4, 50.1]	11.0	[8.7, 13.6]	35.2***	[30.8, 39.7]
Fruits and vegetables (canned)	208	14.4	[9.9, 19.9]	9.6	[6.0, 14.5]	4.8***	[0.1, 11.0]
Meat and seafood	123	72.4	[63.6, 80.0]	7.3	[3.4, 13.4]	65.0***	[55.9, 74.2]
Sweet snacks	43	51.2	[35.5, 66.7]	2.3	[0.1, 12.3]	48.8***	[33.2, 64.4]
Salty snacks	124	66.9	[57.9, 75.1]	8.9	[4.5, 15.3]	58.1***	[48.4, 67.7]
Bread and other cereals	86	69.8	[58.9, 79.2]	25.6	[16.8, 36.1]	44.2***	[30.8, 57.6]
Cheeses	38	47.4	[31.0, 64.2]	23.7	[11.4, 40.2]	23.7*	[0.3, 44.5]
Other^a^	51	17.7	[8.4, 30.9]	3.9	[0.5, 13.5]	13.7*	[0.2, 25.5]
**Ultra-processed foods**	6439	71.0	[69.8, 72.1]	19.9	[19.0, 20.9]	51.1***	[49.5, 52.5]
Yogurt and milk-based beverages	631	92.6	[90.2, 94.5]	26.3	[22.9, 29.9]	66.2***	[62.2, 70.2]
Ultra-processed meat	437	31.1	[26.8, 35.7]	5.3	[3.4, 7.8]	25.9***	[21.4, 30.7]
Breakfast cereals	456	85.8	[82.2, 88.8]	47.8	[43.1, 52.5]	37.9***	[32.3, 43.5]
Seafood	75	89.3	[80.1, 95.3]	9.3	[3.8, 18.3]	80.0***	[70.4, 89.6]
Beverages with non-sugar sweeteners^b^	640	96.7	[95.0, 98.0]	10.8	[8.5, 13.4]	85.9***	[83.2, 88.7]
Sugar-Sweetened beverages^c^	419	75.2	[70.8, 79.2]	19.6	[15.9, 23.7]	55.6***	[50.0, 61.2]
Sweet snacks^d^	1750	68.0	[65.8, 70.2]	20.3	[18.5, 22.3]	47.7***	[44.8, 50.5]
Salty snacks	326	58.9	[53.3, 64.3]	6.8	[4.3, 10.0]	52.1***	[46.2, 58.1]
Packaged bread and tortilla	141	75.2	[67.2, 82.1]	31.9	[24.3, 40.3]	43.3***	[32.8, 53.8]
Cheeses	115	66.1	[56.7, 74.7]	27.8	[19.9, 37.0]	38.3***	[26.3, 50.2]
Ready to eat	165	38.8	[31.3, 46.7]	15.8	[10.6, 22.2]	23.0***	[13.7, 32.3]
Baby food	201	93.0	[88.6, 96.1]	36.3	[29.7, 43.4]	56.7***	[49.2, 64.2]
Soups pastas and creams	197	51.3	[44.1, 58.4]	14.2	[9.7, 19.9]	37.1***	[28.5, 45.6]
Other^e^	886	61.1	[57.8, 64.3]	15.5	[13.1, 18.0]	45.9***	[41.6, 49.6]

Only includes products with claims.

^a^Includes canned beans and prepared salads,

^b^includes beverages sweetened with artificial or natural non-caloric sweeteners or polyols,

^c^Includes nectars, fruit drinks with added sugar, energy drinks, sport drinks and powder to prepare beverages,

^d^Includes candies, sweets, desserts and bakery,

^e^Includes prepared flour bakery, prepared cereals, non-sugar sweeteners and ultra-processed culinary ingredients (margarine, seasonings for meat). The proportion of products with health and nutrition claims in the regulatory scenario was estimated according to the nutrient profile thresholds of the new Mexican food labelling regulation (third stage). The regulatory scenario does not affect the proportion of products with health and nutrition claims of unprocessed or minimally processed foods and culinary ingredients. Chi-squared test, the proportion values were significantly different between current scenario and regulatory scenario: *(*P* < 0.05), ***(*P* < 0.001)

In Table 6, we compared the proportion of products with HNC between the current scenario and the regulatory scenario according to the thresholds for energy and nutrients of concern as per the Mexican regulations. In the current scenario, 94.9% of the products containing non-sugar sweeteners displayed HNC on the FOPL. According to other thresholds, 65.5% of products excessive in saturated fats, 69.4% of products excessive in free sugars, and 70.1% of those excessive in calories displayed HNC. Products that were excessive in calories were 1.40 (*P* < 0.001, 95% CI 1.25–1.56) times more likely to display HNC, compared to products that were not excessive in calories in the current scenario (versus 0.6 times more likely in the regulatory scenario). The Odds Ratio was highest for comparing products that contained non-sugar sweeteners versus those that did not contain such sweeteners (OR 11.67, *P* < 0.001, 95% CI 9.14–14.88). In the current scenario, products that were excessive in saturated fat (OR 0.83, *P* < 0.01, 95% CI 0.75–0.93) and sodium (OR 0.59, *P* < 0.001, 95% CI 0.53–0.65) were less likely to display HNC compared to those products that were not excessive in these thresholds. In the regulatory scenario, we observed a significantly lower proportion of products with HNC for each threshold, mainly for products containing non-sugar sweeteners (18.6% *P* < 0.05, 95% CI 16.7–20.7) and those excessive in sodium (14.3% *P* < 0.05, 95% CI 13.2–15.4). In the regulatory scenario, the odds for displaying HNC were lower compared to the current scenario for each threshold.

**Table 6:** Difference in scenarios for the prevalence of health and nutrition claims on less healthy products

		Proportion of products with health and nutrition claims				Odds Ratio for the use of health and nutrition claims			
	n	Current scenario		Regulatory scenario		Current scenario		Regulatory scenario	
		%	95% CI	%	95% CI	OR	95% CI	OR	95% CI
Excessive in calories	3957	70.1	[68.7, 71.5]	20.6*	[19.3, 21.9]	1.40***	[1.25, 1.56]	0.62***	[0.56, 0.71]
Excessive in free sugars	3392	69.4	[67.8, 70.9]	22.5*	[21.1, 23.9]	0.96	[0.86, 1.08]	0.69***	[0.61, 0.78]
Excessive in saturated fats	2658	65.5	[63.7, 67.3]	19.4*	[17.9, 21.0]	0.83**	[0.75, 0.93]	0.62***	[0.55, 0.70]
Excessive in trans fats	42	64.3	[48.0, 78.4]	23.8*	[12.1, 39.5]	1.08	[0.57, 2.10]	1.21	[0.57, 2.51]
Excessive in sodium	3938	63.6	[62.0, 65.1]	14.3*	[13.2, 15.4]	0.59***	[0.53, 0.65]	0.24***	[0.22, 0.27]
Containing non-sugar sweeteners	1484	94.9	[93.6, 95.9]	18.6*	[16.7, 20.7]	11.67***	[9.14, 14.88]	0.54***	[0.46, 0.62]

Only includes products with claims. The proportion of products with health and nutrition claims in the regulatory scenario was estimated according to the nutrient profile thresholds of the new Mexican food labelling regulation (third stage). Proportion and Odds Ratio (OR) for products with health and nutrition claims according to the current scenario and regulatory scenario. For proportions, *(*P* < 0.05) indicates statistical significant different from current scenario. For Odds Ratio, **(*P *< 0.01), ***(*P* < 0.001) indicates statistical significance

## Discussion

Of the 17,264 products in the Mexican market in 2017, 72% were ultra-processed, 33.8% displayed nutrition claims and 3.4% displayed health claims. About 45% of products had excess sodium and 40% had excess free sugars according to the regulation’s thresholds. The new regulation would prevent about 40% of total products and 50% of ultra-processed food products with claims from displaying health and nutrition claims when the final thresholds go into effect.

The use of HNC on food products in Mexico is consistent with what has been found in other parts of the world; for example, in the UK, 32% [24] of package products carry HNC and 29% in other European countries [36]. HNC could have considerable impacts on diet and thus health. This is because they provide information that may be of interest to consumers [37], especially those interested in improving their health [38].

However, HNC can be used for other purposes such as product marketing [19, 21]. HNC may be more attractive to consumers who may perceive products that have them as being healthier, even if they are not. According to previous studies, foods and beverages with HNC are 75% more likely to be chosen compared to those without these claims [37]. In addition, improving food labelling and HNC regulations are recognized as public health interventions that can improve the food environment and have positive effects on nutrition-related outcomes [39]. Several countries regulate the use of HNC on food products, however, these regulations generally do not include nutrient profiling systems and thus, it is still common to find HNC displayed on less-healthy foods and beverages or ultra-processed products [40]. According to our results, the new Mexican FOPL regulation could improve consumers’ dietary choices, as most less-healthy foods and beverages will be prevented from displaying HNC. This is an opportunity to improve the information on product packaging and avoid ambiguities for consumers; less-healthy foods and beverages will display warning labels but not HNC on FOPL, and conversely, healthier foods may display HNC but not warning labels.

The nutrient profile proposed in the Mexican regulation is based on the PAHO nutrient profile model, which excludes unprocessed foods from evaluation as they are considered healthier products, although there are some exceptions (for Mexico, the consumption of juices and whole fat milk is not recommended [41]). This study showed that a large proportion of ultra-processed food products exceed the thresholds for calories, free sugars, and sodium, so they could be considered as less healthy. In Mexico, the contribution of these products is 30% of the population’s total dietary calories and the consumption of free sugars, saturated fats, and sodium is higher among groups with the greatest intake of ultra-processed foods [10, 42]. In addition, it means that the implementation of the Mexican warning label regulations will reduce the use of HNC mostly on these types of products.

According to a study conducted in the Mexican population, 22% reported using HNC on packaging to select their food and beverages at the point of sale. Until now, there was no strict regulation for the use of HNC in Mexico, so they could be displayed on the packaging regardless of nutritional quality [43]. The regulatory scenario has shown that HNC will be reduced in most less-healthy (ultra-processed) food products, so consumers will be able to make better-informed choices.

In the regulatory scenario, minimally processed or unprocessed foods may continue displaying HNC, so there would be no difference in the proportion of these foods with HNC between both scenarios. However, as previously mentioned, there are food groups not recommended by Mexican food-based dietary guidelines, such as fruit juice, which may still display HNC after the implementation of the regulation [41].

To our knowledge, this is the first regulation that includes a restriction of HNC on FOPL. In addition, it is also the first FOPL regulation to include the use of non-sugar sweeteners as a threshold. There is not enough evidence to describe the long-term effects of consumption of non-sugar sweeteners; however, the recommendations suggest not excluding possible negative effects [44]. However, the use of non-sugar sweeteners is different between countries. In a 2015 study that analyzed the prevalence of non-sugar sweeteners across four countries (Australia, Mexico, New Zealand and the United States), the highest proportion of products containing sweeteners other than sugar was reported for Mexico (11% of all products) [45]. In our study, we found that products with non-sugar sweeteners generally displayed HNC related to the content of calories or sugar (highlighting their absence or low amount). A threshold related to non-sugar sweeteners will likely also prevent unnecessary unhealthy reformulations. In Chile, after the implementation of warning labels, the added sugar content in sugary drinks decreased, but the use of non-sugar sweeteners in these drinks increased [46]. They may display HNC but not warning labels, which could lead to a misperception about nutritional quality.

The Mexican regulation is based on the evidence and recommendations available for the development of effective FOPL [21, 37, 47–49]. However, this regulation has some limitations for HNC, for example, it does not cover health-related ingredient claims and general health claims (considered claims by INFORMAS), which could increase the use of these after regulation. In addition, health claims are restricted on the whole package while nutrition claims not related to excess thresholds can be displayed on the back of packaging. On the other hand, in Mexico there was an interest to regulate the use of health endorsements, a particular type of health claim in which different non-governmental health associations recommend or endorse the consumption of food and beverages for certain groups (for example, “Recommended by the Mexican Association of Pediatrics”). A study conducted in Mexico reported that more than 60% of foods and beverages with health endorsements were classified as less-healthy and endorsements were frequently found in sweetened beverages and sweet snacks; most of the organizations that endorsed these products were professional and independent diabetes and nutrition groups associated with the food industry [50].

### Strengths and limitations

The results of this study provide an approximation to what could be observed after the implementation of the regulation. We also describe the prevalence of products that show HNC, so they could be used as a baseline measure for future evaluations of this regulation. However, the regulatory scenario proposed in this study is conservative, so other possible effects of the new Mexican regulation are not considered, such as changes in the avail of healthier foods (or reformulated foods) that utilize HNC. Another possible effect is an increase in claims not considered in the new regulation on less-healthy foods such as other claims or health-related ingredient claims. These hypotheses may be demonstrated after April 2021, with the mandatory regulation of HNC and other packaging elements (such as characters or promotions) on less-healthy foods.

## Conclusion

The new Mexican front-of-pack labelling regulation is the first in the world to include restrictions on positioning of HNC on FOPL and this study estimated that the new regulation would prevent most less-healthy processed and ultra-processed foods from displaying HNC, in particular those containing non-sugar sweeteners. This is important as a reduction in HNC on less-healthy products may improve the effectiveness of the warning labels for consumers.

## Supplementary Information

**Additional file 1.** Definitions and taxonomy of claims.

**Additional file 2.** Missing data. This document contains the specifications, definitions, examples and taxonomy for the classification of claims, based on the protocols of the International Network for Food and Obesity / non-communicable Diseases Research, Monitoring and Action Support.

## Data Availability

The datasets used and analyzed during the current study are not publicly available. Data are however available upon reasonable request and with permission of the corresponding author.

## Abbreviations

NCDs: Non-communicable diseases
HNC: Health and nutrition claims
FOPL: Front-of-pack labelling
PAHO: Pan American health organization
INFORMAS: International network of food and obesity/non-communicable diseases research, monitoring and action support
