# Supplementary material for: Estimated effects of the implementation of the Mexican warning labels regulation on the use of health and nutrition claims on packaged foods
Source: Int J Behav Nutr Phys Act. 2021 Jun 10;18:76. doi: 10.1186/s12966-021-01148-1 (PMC8194035; doi:10.1186/s12966-021-01148-1)
Supplement: Supplementary file 1 — Additional file 1. Definitions and taxonomy of claims. [file 12966_2021_1148_MOESM1_ESM.docx]

**Additional file 1. Definitions and taxonomy of claims**

Claim types[1]

Health-related ingredient claims

Nutrition claims

Nutrient content claims

Nutrient comparative claims

General health claims

Use of claims

Nutrient and other function claim

Health claims

Reduction of disease risk claim

Other health-related claims

Environment claims

Other claims

| **Table 1. Examples of different types of health and nutrition claims according to the INFORMAS taxonomy** [1,2] | | |
| --- | --- | --- |
|  |  |  |
| Claim | Types of claims | Examples |
| Nutrition claim | Health-related ingredient claim | “Contains 25% orange juice”  “Contains whole grains” |
|  | Nutrient content claim | “Low calories”  “Contains calcium” |
|  | Nutrient comparative claim | “Light”  “Sweetened with stevia” |
| Health claim | General health claim | “Low glycemic index”  “Healthy” |
|  | Nutrient and other function claim | “Ca for strong bones”  “Magnesium for growth” |
|  | Reduction of disease risk claim | “Pediatric heart association”  “Prevents cavities” |
| Other claim | Other claim | “Organic”  “Non-GMO” |
| INFORMAS: International Network for Food and Obesity/non-communicable Diseases Research, Monitoring and Action Support | | |

Definitions[1]

*Nutrition claim:* any representation which states, suggests or implies that a food has particular nutritional properties including but not limited to the energy value and to the content of protein, fat and carbohydrates, as well as the content of vitamins and minerals.

*Health related ingredient claim:* any representation which states, suggest or implies that a food has particular nutritional properties not related to its energy value or to the content of protein, fat and carbohydrates, vitamins and minerals bur related to the content of an ingredient

*Nutrient content claim:* a nutrition claim that describes the level of a nutrient contained in a food [or its energy value]. Includes “Non-addition claims” (any claim that a [nutrient] has not been added to a food, either directly or indirectly. The [nutrient] is one whose presence or addition is permitted in the food and which consumers would normally expect to find in the food”.

*Nutrient comparative claim:* a [nutrition] claim that compares the nutrient levels and/or energy value of two or more foods.

*Health claim*: any representation that states, suggests or implies that a relationship exists between a food or a constituent of that food and health.

*General health claim:* a health claim concerning the general beneficial effects of the consumption of foods or their constituents on health.

*Nutrient function claim:* a [health] claim that describes the physiological role of the nutrient in growth, development and functions of the body. [Although Codex classifies nutrient function claims as nutrition claims it seems more logical to classify them as health claims].

*Other function claim:* health “claims concerning specific beneficial effects to the consumption of foods or their constituents, in the context of the total diet on normal functions or biological activities of the body. Such claims relate to a positive contribution to health or to the improvement of a function or to modifying or preserving health.

*Reduction of disease risk claim:* health “claims relating the consumption of a food or food constituent, in the context of the total diet, to the reduced risk developing a disease or health-related condition.

Claim Content[1,3]

*Health-related ingredient claims*

- Wholegrain
- Fruits/nuts/honey
- Grains/seeds
- Vegetables/plants
- Bacteria/culture/probiotics/prebiotics
- Milk/cream
- Edible oils/oil emulsions
- Cocoa/cacao
- Water

*Nutrient content claims*

- Fibre
- Energy/calories
- Antioxidants/vitamins/minerals/hormones
- Carbohydrates
- Fats
- Sugar
- Protein
- Salt

*Nutrient comparative claims*

- Reduced fat
- More calcium
- Less salt
- Reduced sugar
- Reduced calories
- More fibre
- Reduced carbohydrates
- More protein
- More vitamins/minerals/hormones/antioxidants

*General health claims*

- General e.g. super, healthy
- Low GI/energy density/lower GI
- Digestive health
- Bone health
- Oral health
- Immune health

*Nutrient and other function claims*

- Nutrient + muscle
- Nutrient + bone
- Nutrient + growth
- Nutrient + vision
- Nutrient + energy
- Nutrient + strength
- Nutrient + brain
- Nutrient + nutrient absorption/production
- Nutrient + digestion/bowel
- Nutrient + immunity
- Nutrient + overall health
- Nutrient + blood-related
- Nutrient + oral health

*Reduction of disease claims*

- Heart-related
- Heart Foundation Tick
- Cholesterol absorption
- Glycaemic impact
- Osteoporosis
- Digestive health
- Nutrient absorption

*Other claims*

- Environmental
- Other health-related

References

1. Rayner M, Wood A, Lawrence M, Mhurchu CN, Albert J, Barquera S, et al. Monitoring the health-related labelling of foods and non-alcoholic beverages in retail settings. Obes Rev. 2013;14:70–81.

2. Vandevijvere S, Rayner M. INFORMAS Food Labelling Module - Monitoring health-related labeling and promotional characters/premium offers on foods and non-alcoholic beverages in retail outlets. The University of Auckland: Auckland, New Zealand; 2017. p. 1–61.
