# Supplementary material for: Estimated effects of the implementation of the Mexican warning labels regulation on the use of health and nutrition claims on packaged foods
Source: Int J Behav Nutr Phys Act. 2021 Jun 10;18:76. doi: 10.1186/s12966-021-01148-1 (PMC8194035; doi:10.1186/s12966-021-01148-1)
Supplement: Supplementary file 2 — Additional file 2. Missing data. This document contains the specifications, definitions, examples and taxonomy for the classification of claims, based on the protocols of the International Network for Food and Obesity / non-communicable Diseases Research, Monitoring and Action Support. [file 12966_2021_1148_MOESM2_ESM.docx]

Additional File 2. **Missing data**

For the purposes of this study, we used the information on claims, the list of ingredients, and nutrient declaration available on food and beverage packaging labels. For products with missing data, the partial omission method was used to exclude them from the analyses that required that information.

Information on the use of claims was available for all products. Information on the list of ingredients was available for 97.0% of the products analyzed; this data was required to classify foods and beverages according to the NOVA food classification system and to identify added nutrients of concern, such as free sugars, fat and sodium and additives like non-sugar sweeteners. According to the specifications of the previous Mexican food labelling regulation, single ingredient foods that do not include additives could exclude the list of ingredients [1]. Foods that did not report the list of ingredients (3%) were classified according to the extent and purpose of food processing. For example, packaged fruit and sugar were classified as unprocessed or minimally processed foods and processed culinary ingredients (respectively), and canned fruits and sugar-sweetened beverages were classified as processed and ultra-processed foods, respectively.

The information on nutrient declaration (serving size, calories, fats, sodium and total sugars) was available for 99.8% of the products and was used to evaluate the nutritional quality of foods and beverages, mainly for processed and ultra-processed foods. Products that did not include data on nutrient declaration were excluded from the nutritional quality analysis (0.2% from ultra-processed foods). However, according to the Mexican food labelling regulation before 2020 [1], the declaration of free sugars and trans fats was not mandatory. Information for free sugars was available for 480 products, so these were estimated according to the algorithms proposed by the Pan American Health Organization Nutrient Profile Model indicated in Table 1 [2].

Information for trans fats was available for 5,113 (29.6%) products. The estimation of the amount of trans fats is more sensitive to errors for two main reasons [1,3]. Firstly, because they are found in small quantities and secondly, because the regulation allowed rounding amounts in the nutrient declaration (e.g. fat components can be rounded at 0 g when the content is <0.5 g per serving). Then, it was common to find that most of the products with ingredients considered as sources of trans fats like partially hydrogenated vegetable oil reported 0 g of trans fat. Therefore, we decided to use the information available on 5,113 products and not make imputations for this nutrient. This decision leads to an underreporting of the proportion of products that can be classified as excessive in trans fats. However, other studies conducted in Mexico estimated that less than 1% of the products would be classified as excessive in trans fats [4]. This is reflected in Table 6; trans fats are the nutrient of concern that would have the least reduction in the prevalence of health and nutrition claims.

**Table 1. Methods to estimate the content of free sugars proposed by the Pan American Health Organization Nutrient Profile Model** [2]

| **If the manufacturer declares…** | **Then estimated free sugars equal…** | **Examples of products** |
| --- | --- | --- |
| 0 g of total sugars | 0 g | Canned fish |
| Added sugars | Declared added sugars | Any product that declares added sugars |
| Total sugars, and the product is part of a food group with no or a minimal amount of naturally occurring sugars | Declared total sugars | Regular soft drinks, sport drinks, sweet biscuits, breakfast cereals, chocolate |
| Total sugars, and the product is yogurt or milk, with sugars in the list of ingredients | 50% of declared total sugars | Flavored milk or yogurt |
| Total sugars, and the product is a processed fruit item with sugars in the list of ingredients | 50% of declared total sugars | Fruit in syrup |
| Total sugars, and the product has milk or fruit in the list of ingredients | 75% of declared total sugars | Cereal bar with fruit |

**References**

1. Secretaría de Salud. NOM-051-SCFI/SSA1-2010, Especificaciones generales de etiquetado para alimentos y bebidas no alcohólicas preenvasados-Información comercial y sanitaria [Internet]. D. Of. la Fed. 2010. p. 1–31. Available from: http://dof.gob.mx/nota_detalle.php?codigo=5137518&fecha=05/04/2010

2. Pan American Health Organization. Nutrient Profile Model [Internet]. Washington, DC; 2016 [cited 2021 Apr 20]. p. 6. Available from: http://www.euro.who.int/en/health-topics/disease-prevention/nutrition/publications/2015/who-regional-office-for-europe-nutrient-profile-model-2015

3. Ricardo CZ, Peroseni IM, Mais LA, Martins APB, Duran AC. Trans fat labeling information on Brazilian packaged foods. Nutrients. 2019;11:14.

4. Contreras-Manzano A, Jáuregui A, Velasco-Bernal A, Vargas-Meza J, Rivera JA, Tolentino-Mayo L, et al. Comparative analysis of the classification of food products in the mexican market according to seven different nutrient profiling systems. Nutrients. 2018;10.
